# Changes in Mechanical Properties of Fabrics Made of Standard and Recycled Polyester Yarns Due to Aging

**DOI:** 10.3390/polym15234511

**Published:** 2023-11-23

**Authors:** Ines Katić Križmančić, Ivana Salopek Čubrić, Vesna Marija Potočić Matković, Goran Čubrić

**Affiliations:** 1 2 K ideja d.o.o., 10000 Zagreb, Croatia; ikatickri@ttf.unizg.hr; 2 University of Zagreb Faculty of Textile Technology, 10000 Zagreb, Croatia; marija.potocic@ttf.unizg.hr (V.M.P.M.); goran.cubric@ttf.unizg.hr (G.Č.)

**Keywords:** yarn, fabric, aging, material, polymer, polyester, elastane, recycled, elongation at break, force, surface morphology, moisture management

## Abstract

Over the years, the demands on the durability and quality of polyester fabrics used for sportswear have increased, as these fabrics contribute to athletes’ performance. At the same time, the use of recycled polyester material is increasingly being promoted for environmental reasons. This study focused on investigating the properties of standard and recycled polyester fabrics before and after aging according to the developed aging protocol. The surface morphology, thickness, elongation at break, force at break, bursting force, mass loss due to abrasion and moisture management of the fabrics were tested. The results showed that the aging process had no influence on the surface changes in the fabrics. More specifically, there were neither surface cracks on the fibre surface nor chemical changes. The highest decrease in force at break for standard polyester fabrics with elastane was up to 26%, and up to 15% for fabrics made of recycled polyester. The loss of mass due to abrasion was greater for recycled polyester than for standard polyester fabrics. The average ability of the fabrics to absorb moisture decreased by up to 23% after aging, while the wetting time increased by up to 30%, with the highest increase observed in recycled fabrics.

## 1. Introduction

Playing football is possible both as a leisure activity and actively. In active football, there are beginners, cadets, juniors, seniors and veterans. The typical football activities depend on the level of football practice, as well as the position in which the player plays (striker, midfielder, defender and goalkeeper). Fitness training, tactics and recovery training are common. The fabrics for football sportswear are usually made of polyester and elastane and, more recently, recycled polyester fabrics [1,2]. They are exposed to wear and tear, the effects of perspiration, aging through care and maintenance and atmospheric conditions. Changes in the mechanical properties of polyester materials, including sportswear fabrics, can be caused by temperature, sunlight (UV), humidity, rain and wind and pollutants [3,4]. Wind with various particles can have an abrasive effect on the surfaces of the materials. Fluctuations in temperature and humidity lead to uneven stress and surface cracking in polyester. In addition, UV light has a chemical effect on polyester through photodegradation. The amount of UV light depends on latitude, season and cloud cover. All these factors, together with various pollutants, make the aging process of football sportswear complex [3,4].

Outdoor weathering, i.e., exposure to the natural environment, is still the standard for material aging, although test methods and instruments have been developed to accelerate aging [5]. Accelerated aging uses enhanced conditions such as heat, humidity and sunlight in environmental chambers to accelerate the normal aging processes, or accelerated mechanical testing [5]. Natural or accelerated weathering of polyesters leads to scission, i.e., breaks and splits in the polymer chains centred on the ester linkages. Polyesters and many other polymers hydrolyse in the presence of water [3,6]. The hydrolysis of polyesters is usually carried out with the addition of a base to accelerate the reaction. The process has advantages and disadvantages: biodegradability, but also the deterioration of mechanical properties.

Several studies on the change in the mechanical properties of polyester during aging relate to technical textiles. In one study [7], high-strength polyester yarns with different linear densities for the reinforcement of conveyor belts were aged at temperatures of 140, 160, 200 and 220 °C for a duration of 6, 12 and 35 min. It was confirmed that the aging parameters significantly affect the mechanical and surface structural properties when the exposure temperature is 220 °C. The results of the mechanical properties and aging behaviour tests of palm/polyester composite for automotive industry showed that this composite has excellent tensile strength and abrasion resistance, and the FTIR and SEM data after the climate aging test confirmed the durability [8]. In another study [9], polyester composites intended for sewer rehabilitation were subjected to artificial aging with heat and water. The samples aged in water were stored in ventilated ovens at 40 °C, 60 °C and 80 °C or kept at room temperature (20–22 °C). Half of the samples were aged in air only. It was found that aging in water at high temperatures resulted in greater effects on the material than aging under dry conditions [9]. Accelerated aging of polyester multifilament yarns containing nanoparticles or microparticles was carried out in a climatic chamber at 50 °C, 50% Rh and UVA irradiation of 1.4 W/m^2^ between 21 and 170 days [10]. The results showed that there was a deterioration in the thermal and mechanical properties of all yarns, possibly due to the scission of the polymer chains that make up the polymer matrix [10].

Several studies have been carried out on polyester textile fabrics intended for the clothing industry. After three months of natural outdoor aging, the water vapour resistance of polyester textiles coated with polyurethane decreased, which correlates with the reduced thickness of the textile. The average reduction in water vapour resistance after aging was 11.4% in summer and 16.7% after the winter season [11]. There was a partial degradation of the polyurethane layer, which was not related to the deterioration of the polyester fabric substrate [11]. As for outdoor aging compared to pool aging up to 21, 42 and 63 h, the deterioration of the surface is more pronounced after outdoor aging, confirming the effect of UV radiation on the fibrillation of polyester and polyamide [12]. All measured properties, tensile strength, drying and fluid transfer, with the exception of elongation during indoor aging, confirm the influence of water and chlorine and especially solar radiation on the degradation of polyester [12].

The behaviour of recycled polyester is less studied, although there are more and more commercially available products made from recycled polyester, including sportswear. One study shows that the final properties of the recycled polymers differ from the properties of the samples before reprocessing [13]. During their lifetime, polymers are exposed to various environmental stresses that can greatly alter their chemical and molecular structure and morphology, as well as further recycling [13]. In another study, the recyclability of the four plastics most commonly found in the sea (nylon, PE, PET and PP) was investigated. They were exposed to UV radiation in seawater for 6.5 months. The properties of all materials were affected, resulting in inadequate quality of the recycled material compared to virgin material [14].

A fibre that is often added to polyester to improve the elastic properties of sportswear is elastane. The presence of elastane has a significant influence on the material properties before and after aging. Elastane includes polyetherurethane or polyterurethane polymers. It is known that polyester urethanes are at risk of hydrolysis with aging [15], which affects mechanical properties. The shortcomings of elastane also lie in its chemical resistance and temperature stability, which can lead to fibre degradation and loss of elasticity [16]. Further research into the aging of sportswear fabrics with elastane is a necessity for product quality.

Despite the increased interest in the development of highly functional polyester fabrics for the production of sportswear, to our knowledge scientists have not yet studied the aging of polyester fabrics for sportswear in depth. In particular, this concerns the development and testing of different protocols defined considering a specific group of fabrics for a single use. Knowing the behaviour of the material and its properties due to aging is important because it can affect comfort during sports competitions, reduce durability during maintenance and increase the possibility of the material tearing in direct contact between two football players.

In the previously published study [17], steps were taken to develop a specific procedure for the aging of polyester sportswear fabrics. The aging process was correlated with 12 training sessions (1 month) and 24 training sessions (2 months) of football training. It was emphasized that future studies should focus on a longer aging period. In this study, a new aging protocol is established with a longer exposure period corresponding to 1 month to 3 months of training. After aging, a series of tests were performed on the fabrics to determine the mechanical and comfort properties of the fabrics, such as elasticity and moisture management, which are very important for the well-being of the athlete [18,19]. The focus is on comparing the behaviour of standard polyester and recycled polyester to determine the extent to which recycled polyester can be an adequate substitute for standard polyester when exposed to severe aging.

## 2. Methods and Materials

### 2.1. Material

For this study, a number of representative materials were selected that are predominantly used in the manufacture of football sportswear. These were knitted fabrics made of standard polyester with elastane added and fabrics made of recycled polyester without elastane. The aim was to investigate the changes in the properties of the two material groups as a result of aging. All fabrics are made of polyester filament yarns (more precisely from polyethylene terephthalate), which are twisted in the Z-direction and have an average thickness of 1.1 mm. During production, the elastane yarn is plated into every second course of stitches. The fabrics were not dyed. The fabric ID and the description can be found in Table 1.

### 2.2. Aging Protocol

When defining the aging protocol, it was important to include a wide range of influencing parameters that affect aging in order to simulate as accurately as possible the environment and influences under which the material is expected to perform. In this study, the protocol for the aging of fabrics used for football sportswear is defined following a survey of professional athletes, additional interviews and information from the literature reviewed. The survey was conducted using an online questionnaire approved by the Ethics Committee of the University of Zagreb Faculty of Textile Technology. A total of 86 Croatian football players were included in the survey. The survey was created and processed using SurveyTool v8.4 (3S, Stockholm, Sweden).

The protocol comprised a series of steps, i.e.;

- Definition of the target group;
- Determination of the training conditions of the target group;
- Selection of materials;
- Determination of the aging factors;
- Determination of the aging processes;
- Determination of the test methods.

The aging was carried out according to the aging protocol (details shown in Table 2). The target groups were football players in the junior and senior categories. Juniors and seniors train three times a week. The duration of each training session averages 2 h, resulting in a total of 24 h of training per month. Converted into minutes, this results in a total time of 1440 min per month. The cycle with exposure of fabrics, corresponding to one month of training, is labelled A1, while the cycle corresponding to three months of training is labelled A2. The unexposed cycle is referred to as A0.

During the 120-min individual training session, the fabric for athlete’s clothing was exposed to sweat for approximately 105 min, i.e., 1260 min during the entire training month. As the training took place outdoors, the fabrics were also exposed to other external factors such as the sun. Usually, the sports jersey must be washed after every single training session. The required number of washes for each specified aging cycle and the average washing time are indicated in Table 2.

The fabrics were exposed to weathering, simulating typical use by football players. This involved static outdoor weathering to which artificial sweat was added. The weathering was conducted during the summer season (July–August) at the coordinates 45°48′055.4364″ N, and 15°57′059.6448″ E. The average air temperature was 31 °C (with a range of 28 °C to 35 °C and a coefficient of variation of 7.36%). The average UV index was 8 (with a range of 6 to 9 and a coefficient of variation of 14.49%). The average humidity was 58% (between 48% and 64%, with a coefficient of variation of 17.27%). The average wind speed was 7.5 km/h (6 km/h to 9 km/h; coefficient of variation 18.37%) and the air pressure was 1015 mbar (varied from 1008 mbar to 1029 mbar; coefficient of variation 0.66%). The overall air quality was 81 AQI (65 AQI to 90 AQI; coefficient of variation 11.69%). All data were monitored by the European Meteorological Center ECMWF using model weather forecasts HRES [20]. The duration of exposure of the fabric to sun and sweat is given in Table 2. A dilution of acid sweat powder (AS) with a pH of 5.5, prepared in accordance with BS EN ISO 105-E04 [21], was added to the samples 15 min after the start of the exposure to the sun. After each 2-h simulated training session, the fabric was washed at 30 °C using the ECE detergent (without phosphate and optical brighteners). Washing was carried out in accordance with the EN ISO 6330: 2012 standard [22]. After each wash cycle the fabrics were air-dried in the shade (i.e., they were not exposed to the sun during air-drying).

### 2.3. Characterization Methods

This study focused on the following aspects: surface morphology characterization, thickness, elongation at break, force at break, bursting force, moisture management and mass loss due to abrasion.

#### 2.3.1. Surface Morphology Characterization

A Dino-Lite Edge AM7915MZT digital microscope (Dino-Lite, Almere, The Netherlands) was used to assess the topography of the selected fabrics. The samples, both non-aged (A0) and aged (A1, A2), were conditioned in a standard atmosphere (i.e., at an air temperature of 20 ± 2 °C, and a relative humidity of 65 ± 5% [23]). The images of the samples were taken at 150× magnification. DinoCapture 2.0 software was used to observe the appearance of the surface of the fabrics, and the shape of the stitches forming the structure of the knitted fabric. It was also used to measure the length of the horizontal inner space of the stitches (L_in-avg_). The microscope FE-SEM (Tescan, Brno, Czech Republic) was also used to observe the surface of the fabric. The samples were coated with chromium, and the acceleration voltage during the measurements was 5 kV.

#### 2.3.2. Thickness

The thickness was measured according to the principles described in [24] using a DM-2000 thickness meter (Wolf Messtechnik GmbH, Freiberg, Germany). The pressure of the device was set to 1 kPa, and 10 measurements were taken. The thickness of the fabric was given as the average of the measurements.

#### 2.3.3. Elongation at Break and Force at Break

The elongation at break and force at break were measured with the Statimat M tensile tester (Textechno, Mönchengladbach, Germany) at a constant elongation rate, according to ISO standard [25]. The test speed was set to 100 mm·min^−1^, and the load cell was 1000 N. The gauge length was set to 100 mm, with an error of ±1 mm. For testing are prepared rectangular specimens in dimensions 5 × 20 cm. The tests were carried out in wales and courses direction. The average result was expressed as the mean value of 5 measurements.

#### 2.3.4. Bursting Force

A burst tester (Apparecchi Branca, Milano, Italy) was used to measure the bursting force. The device is equipped with a steel ball with a 25.40 ± 0.005 mm diameter. The tests were carried out in accordance with the ASTM D3787 standard [26]. According to the method used, a force was applied to the sample, which caused the sample to burst. The probe was circular, with a diameter of 50 ± 1 mm. The number of samples for each fabric was 5. The force was recorded at the moment of break.

#### 2.3.5. Moisture Management

The parameters describing the fabric moisture management were tested using the Moisture management tester, model M290 (SDL Atlas, Rock Hill, SC, USA). The measurements were performed according to the AATCC TM 195-2021 standard [27]. The wetting time, absorption rate and spreading speed were measured. The wetting time (WT) was measured on the top surface of the fabric (WTT), and on its bottom surface (WTB). WT is defined as the time required to wet the top and bottom of the fabric, measured from the start of wetting. The absorption rate (AR) is the average moisture absorption capacity of the surface (top side—TAR, and bottom side—BAR). It is represented as the slope of a curve between the point at which the sample begins to wet and the maximum point on the water content vs. time graph. The spreading speed (SS) is the cumulative spreading velocity. The device measures the top spreading speed (TSS), and the bottom spreading speed (BSS).

The overall moisture management capability (OMMC) is an index calculated from the values of the measured parameters, i.e.,

(1) $$OMMC=C_{1}\cdot {AR}_{B}+C_{2}∙R+C_{3}∙{SS}_{B}$$

where the *OMMC* is overall moisture management capability; the *AR_B_* is absorption rate; the *R* is one-way transport capability; the *SS_B_* is spreading speed, and *C*_1_, *C*_2_ and *C*_3_ are the weighting values for the listed properties.

#### 2.3.6. Abrasion

The AquAbrasion Tester (James Heal, Halifax, UK) was used to measure the abrasion of materials. This is a special device based on the Martindale principle, developed for testing materials exposed to different weather conditions. The tester is compatible with the international standard [28]. According to the standard, the samples were cut to a diameter of 38 ± 5 mm. The standard wool fabric (with the properties defined in the mentioned standard) was used for abrasion. The sample was clamped in the sample holder of the tester and subjected to a load of 9 kPa. It was rubbed against the abrasive medium in a motion following the Lissajous curve. The mass loss method was used to estimate the abrasion resistance of the tested fabrics. Accordingly, each sample was weighed before the test and after a certain number of test cycles (500, 1000, 2500 and 7500). A KERN ALJ—220 analytical balance (Kern & Sohn GmbH, Balingen, Germany) with an accuracy of +/− 0.001 g was used for weighing.

#### 2.3.7. Fourier Transform Infrared Analysis

Fourier Transform Infrared Analysis was performed using the Perkin Elmer spectrometer (Perkin Elmer Inc., Waltham, MA, USA). Ten scans with a resolution of 4 cm^−1^ in a range from 4000 to 450 cm^−1^ were performed for each sample.

## 3. Results and Discussion

### 3.1. Results of the Surface Morphology Characterization

**Figure 1:**
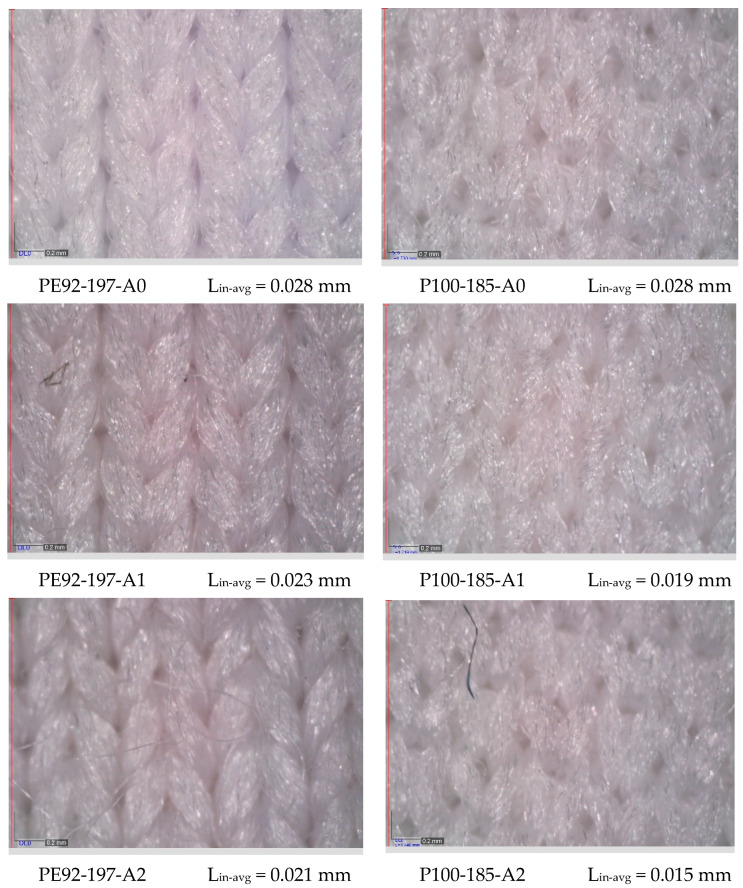
Microscopic images of standard and recycled polyester fabric in stages A0, A1 and A2.

Regarding the surface morphology of investigated fabrics, the differences between the fabrics made of standard and those made of recycled polyester are observed. To explain the differences, images of one standard polyester fabric (PE92-197) and one recycled polyester fabric (P100-185) are shown in Figure 1. Before aging, the differences between fabrics made of standard polyester and recycled polyester yarn are clearly visible. The structure of the stitches (i.e., the basic units that form the knitted fabric) of standard polyester fabrics is strictly vertically aligned, and the units are clearly visible. In contrast, the structure of recycled polyester fabric does not follow a clear vertical orientation, and the stitches are not uniformly shaped. In addition, the number of protruding fibres increased, which affected the increase in the average diameter of the yarn. The size of the internal cavity within the stitches is difficult to distinguish. In both standard and recycled fabrics, the aging process caused the increase in foreign particles that have become entangled in the fabric structure. Moreover, there was the increase in protruding fibres in higher length classes from the structure of the fabrics. The structure of the recycled fabric and the shape of the stitches are even less recognizable. As can be seen from Figure 1, the average values of the measured length of the horizontal inner space of the stitches (i.e., L_in-avg_) decrease after the fabrics are exposed to the sun. More specifically, L_in-avg_ is 0.028 mm for the non-aged fabrics PE92-197 and P100-185. The values decrease to 0.021 mm and 0.015 mm after aging A2. Considering the length of exposure, it can be concluded that longer exposure causes a further decrease in the length of the inner space of the stitches. The reason for this can be found in the shrinkage of all fabrics. The decrease in the length of the inner space of the stitches and fabric shrinkage is again more pronounced in recycled fabric. This observation is consistent with the conclusions of the study on the effect of heat treatment on the physical properties of nonwovens [29]. This study confirmed that the shrinkage of the recycled polyester nonwovens was higher than that of standard polyester, regardless of the heat temperature, and exposure duration.

**Figure 2:**
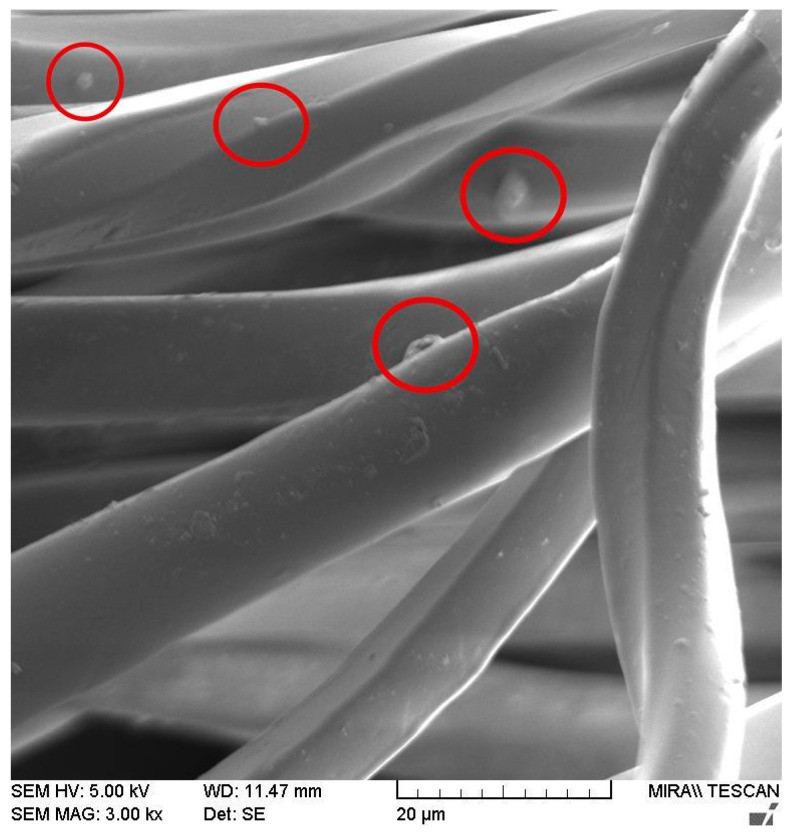
SEM image of aged standard polyester fabric.

Figure 2 shows a representative SEM image of the aged standard polyester fabric at a magnification of 3.00 kx. The results of the SEM analysis showed no age-related surface cracks on the fibre surface. However, the image of the aged fabric (Figure 2) shows an accumulation of particles on the fibre surface (marked in red) that were not present prior to material aging. The water with an average hardness of 15° dH was used to wash the fabric in this experiment. Therefore, it is very likely that these particles are limescale or detergent residues that were not completely removed during the washing process. The SEM images (of both standard and recycled polyester) showed no noticeable differences after aging.

### 3.2. Results of Thickness Testing

**Figure 3:**
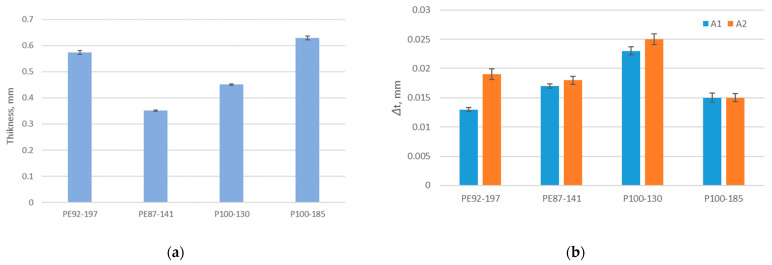
The thickness of fabrics (**a**) thickness of non-aged fabrics, (**b**) change in thickness (Δt) due to aging (A1 and A2) in comparison to thickness of non-aged fabrics.

The results of the thickness test of non-aged fabrics are shown in Figure 3a. The change in thickness of fabrics due to aging is shown in Figure 3b. The results show that aging of all tested fabrics in both aging protocols caused an increase in thickness compared to the thickness of the unexposed fabric (the *p*-values are higher than 0.05, which means that there is no statistically significant difference). These results complement the behaviour of the materials observed in the previous section, and confirm that aging affects the shrinkage of the materials. This increase in thickness can also be explained by the increase in the number of protruding fibres. It can be seen that aging A2 caused an additional increase in the thickness of the fabrics made of standard polyester compared to the increase after A1. In contrast, the increase in thickness of the fabrics made of recycled polyester is the same after A1 and A2 (i.e., in both cases the increase is 0.015 mm).

### 3.3. Results of Elongation and Force at Break Testing

**Figure 4:**
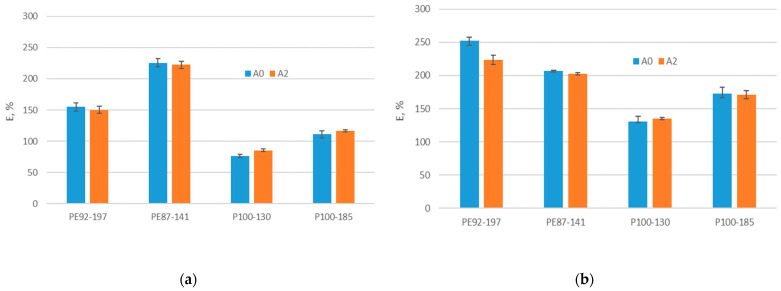
Elongation at break of non-aged (A0) and A2 aged fabrics in the direction of (**a**) wales, (**b**) courses.

As the knitted structure can elongate, it can better adapt to the human body and respond appropriately to the stretching that occurs during sporting events. Therefore, it is desirable to know what happens to the elongation of the fabric subjected to aging. Figure 4 shows the elongation at break for non-aged and A2-aged fabrics (three months of training). The tests were carried out both in the direction of wales and courses. It is apparent that recycled polyester fabrics have a much lower elongation at break than standard polyester fabrics with elastane. The results showed that the elongation at break of standard polyester fabrics with elastane is lower after aging, and this is statistically significant for the direction of wales only (*p*-value is 0.04). For recycled fabrics, the situation is reversed. The observed parameter on average increases, except for the sample P110-185. There are no statistically significant differences between the elongations at break of the recycled fabrics (*p*-value is greater than 0.05). The results obtained show that standard polyester fabrics with elastane still perform better in terms of elongation at break, despite the fact that their elongation at break decreases after three months of aging.

**Figure 5:**
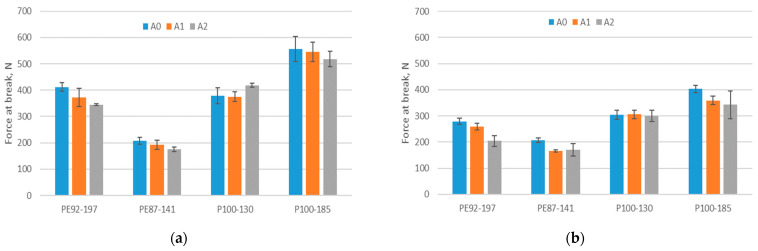
Force at break of non-aged and aged fabrics in the direction of (**a**) wales, (**b**) courses.

**Figure 6:**
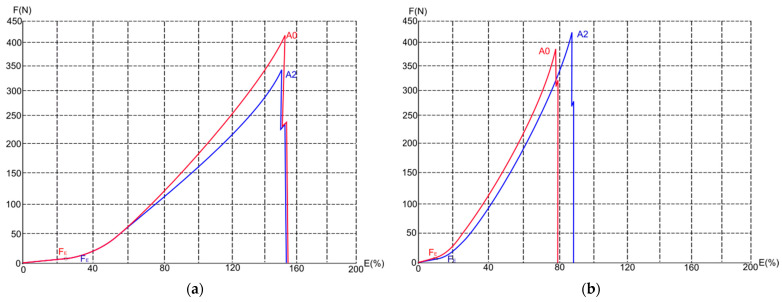
The F/E curves in the conditions A0 and A2 for the samples (**a**) PE92-197; (**b**) P100-130.

The results of the measured force at break are shown in Figure 5. On average, the force at break of fabrics decreases with aging. The highest decrease for standard polyester fabrics with elastane is up to 26%, while it is up to 15% for fabrics made of recycled polyester. There are statistically significant differences between forces at break for the direction of courses (*p*-value between A0 and A1 samples is 0.04; *p*-value between A0 and A2 samples is 0.03). There are no significant differences for the direction of the wales (i.e., *p*-value ≥ 0.05). Figure 6 shows a plot of the specific force–elongation curves (i.e., F/E curves) for samples PE92-197 and P100-130 under conditions A0 and A2. As can be observed, standard polyester material with elastane has a much higher elongation at break than recycled polyester material. It is most probably because of the presence of elastane. In addition, the force at the maximum point of elasticity (F_E_) is higher for standard polyester fabric with elastane than for recycled polyester fabric. The force at break for both fabrics is similar, but at this force, the standard fabric with elastane stretches almost twice as much as the recycled fabric, which does not contain elastane in its structure.

### 3.4. Results of Bursting Force Testing

**Figure 7:**
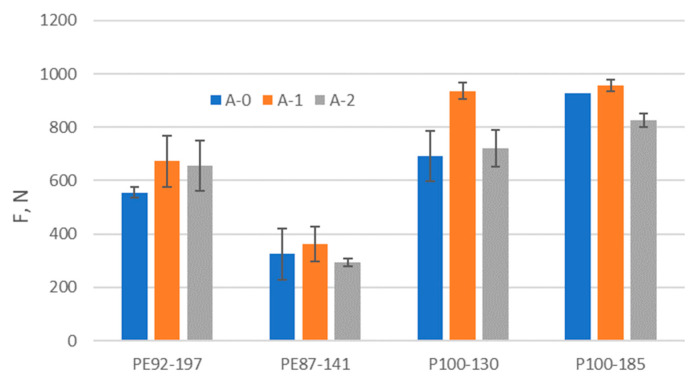
The bursting force.

The results of bursting force testing can be found in Figure 7. It can be seen that the bursting force of all tested samples increased after aging A1. This observation is consistent with the conclusions on the retention of excellent strength of polyester composites reported in the previous study [8]. The increase in bursting force could be related to the increase in thickness observed in all exposed samples. Indeed, the thicker material could provide a higher resistance to bursting with a steel ball, which is reflected in the higher values of bursting force.

After the aging A2, the bursting force decreases for all samples compared to the results of the aging A1. Nevertheless, samples PE92-197 and P100-130 retain higher values compared to the non-exposed samples. The results obtained show that, despite the aging process, samples PE92-197 and P100-130 respond better to the bursting force to which the fabrics are frequently exposed in contact sports such as football. In contrast to the study [14], in which the properties of recycled fabrics exposed to UV radiation and seawater were strongly negatively affected, the results do not show the insufficient quality of the observed properties of recycled fabrics in terms of bursting force.

### 3.5. Moisture Management Test Results

**Figure 8:**
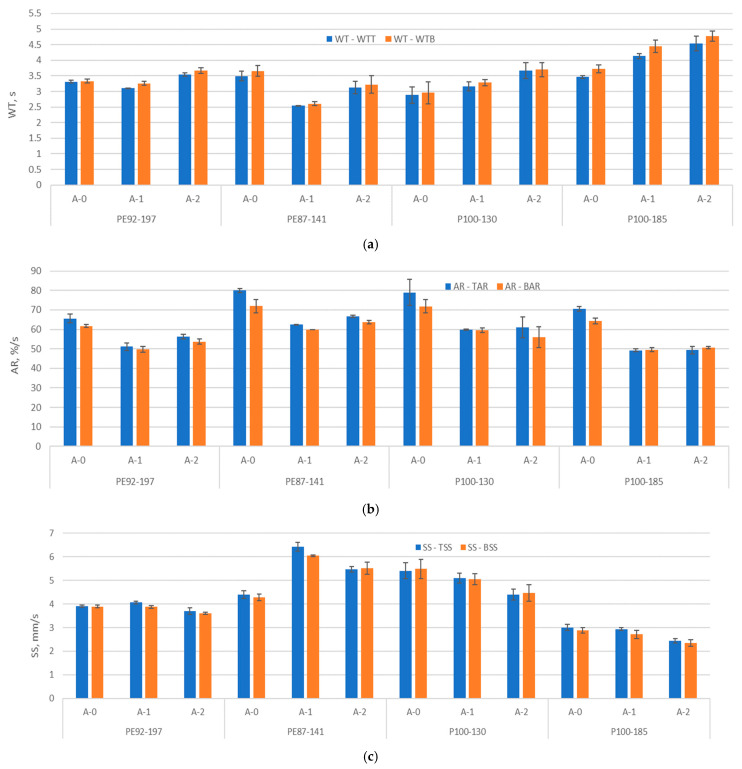
The moisture management results, (**a**) wetting time—WT (WTT—top, WTB—bottom); (**b**) absorption rate—AR (TAR—top, BAR—bottom); (**c**) spreading speed—SS (TSS—top, BSS—bottom).

The moisture management test results, including WT, AR and SS, are shown in Figure 8. The results show that the wetting time (WT) increased on both surfaces (bottom—WTB and top—WTT) for all samples after A2 aging, except for sample PE87-141. A significant increase in wetting time (30% on average) can be observed for the samples made of recycled polyester. The increase in wetting time can be linked to the increase in fabric thickness caused by the aging. The average ability to absorb moisture (AR) on the surface of the fabric on both sides (top—TAR and bottom—BAR) decreased by an average of 23% for all tested samples after exposure to aging A1 and by an average of 20% after exposure to aging A2. This can be related to the shrinkage of the fabric, and the decrease in the length of the inner space of basic units (observed in Section 3.1). The spreading speed rate decreased for all samples, except for PE87-141. The results confirm the strong negative correlation between the values of WT (both WTT and WTB) and SS (both TSS and BSS) with correlation coefficients −0.86727, −0.85289, −0.88493 and −0.87779 (Table 3).

**Figure 9:**
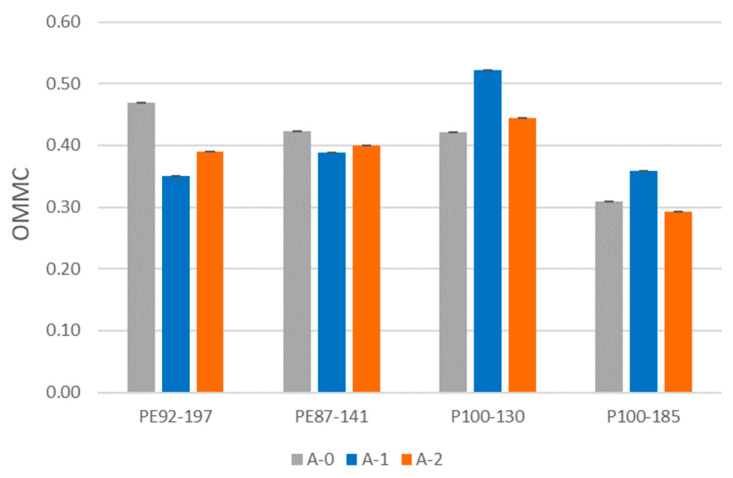
The total moisture management capability (OMMC) of fabrics.

**Figure 10:**
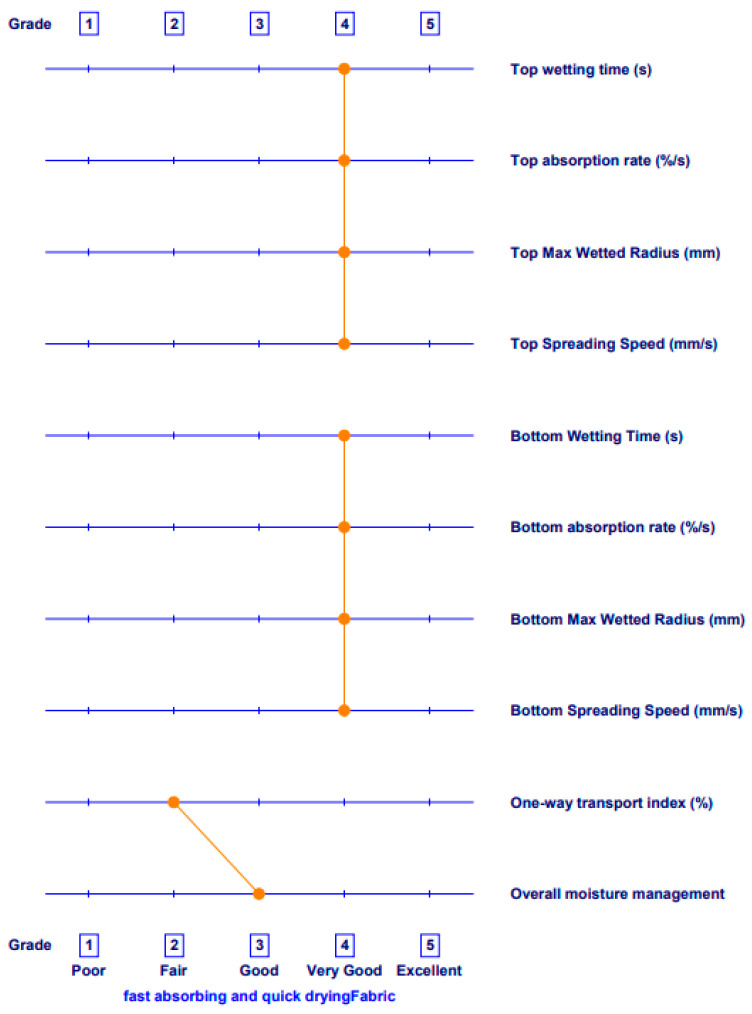
The illustration of the fingerprint of sample PE92-197, non-exposed (i.e., A0).

The results of the changes in the OMMC index after exposure to aging are shown in Figure 9. The illustration of the fingerprint of the polyester fabric PE92-197 before aging is shown in Figure 10. The strongest increase in the index, after the A1 aging process, was observed for the recycled polyester fabrics. For the standard polyester fabrics, it was lower than before the aging. Comparing the results of the index before aging, and after aging A2, a decrease was observed in all samples. The exception is a sample P100-130 with a slight increase in OMMC index.

All samples, both before and after aging, were characterized as fast-absorbing and quick-drying. However, the changes in the OMMC value after aging were not statistically significant for all fabrics tested (*p*-value was 0.755). For the OMMC values, which were recorded on a scale of 1 to 5, changes were visible before and after the aging process. Namely, for the samples made of standard polyester (PE92-197 and PE 87-141), grade 3 was assigned before aging. After the A-1 aging it was downgraded to 2. The grade did not change after either aging process. for two samples made of recycled polyester.

### 3.6. Results of Abrasion Testing

**Figure 11:**
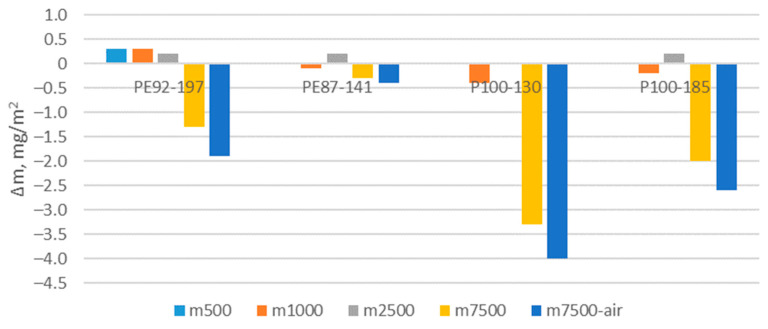
Changes in mass per unit area of A2 aged fabrics due to abrasion in comparison to the mass of non-abraded aged fabrics.

The abrasion test was carried out on fabrics after aging A2 only. The results are shown in Figure 11. The changes in mass per unit area due to abrasion are compared to the mass per unit area of the non-abraded fabrics. The comparison was made after 500, 1000, 2500 and 7500 cycles. After 7500 abrasion cycles, the samples were additionally exposed to airflow to remove residual fibre particles that had been abraded from the structure of the fabric. The mass per unit area of some samples showed a slight increase after 500–2500 abrasion cycles, which is due to the increase in foreign particles/fibres that have penetrated the fabric structure. After 7500 abrasion cycles, a significant loss of mass can be observed, especially in aerated samples. For aerated samples, the foreign particles are removed with compressed air. The test results for the PE87-141 sample show a particularly low mass per unit area loss, and a correspondingly high resistance to the abrasion process. The loss of the mass per unit area is particularly pronounced for the fabrics made of recycled polyester (P100-130 and P100-185). These results indicate a significantly lower quality of these two fabrics when they are exposed to abrasion. The latter is likely to affect the visual perception of the fabric as well as the touch properties. This may result in a negative impact on the perception of comfort. The results are in accordance with the results of the study conducted by Yaping et al. [30]. The researchers used the AquaAbrasion tester to investigate the influence of abrasion of synthetic textiles on the formation of microplastic fibres and fibrils. Yaping et al. showed that abrasion of material can release ten times more microplastic fibres (MPF) into the environment than a single washing process.

### 3.7. Results of Fourier Transform Infrared Analysis

**Figure 12:**
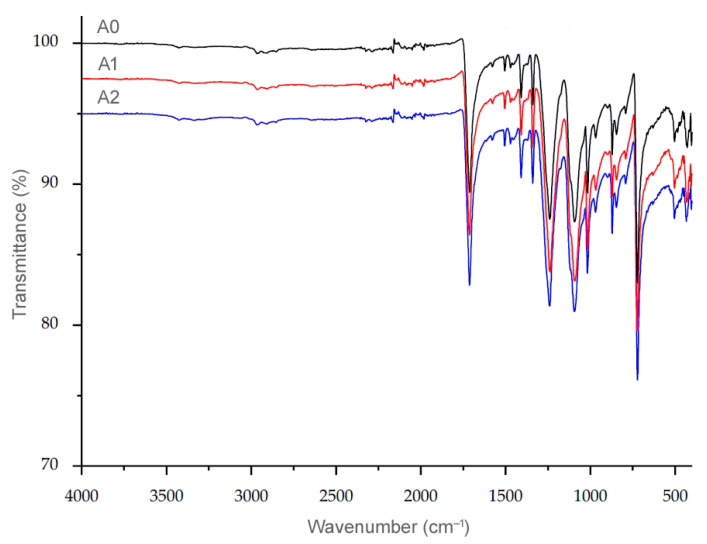
IR spectra for polyester fabric (A0, A1 and A2).

In order to investigate possible chemical changes in the observed fabrics, an FTIR analysis is carried out. Figure 12 shows a typical plot for the fabrics studied, in which the spectral data of the non-aged and aged fabrics are superimposed.

The results indicate that no chemical changes have taken place during the aging of both the standard and the recycled polyester, as the spectra of the initial fabric (i.e., the non-aged fabric) cannot be distinguished from the spectra of the aged fabrics.

## 4. Conclusions

This study investigates the changes in the mechanical properties of fabrics due to aging. It focuses on the behaviour of knitted fabrics made of recycled polyester and standard polyester with added elastane. The results show that the aging of all investigated fabrics influenced the increase in thickness compared to the thickness of the unexposed fabric but without statistical significance. The force at break for the tested fabrics decreased due to aging, and this decrease is statistically significant for the direction of wales. This behaviour of aged fabrics can increase the likelihood that the fabric will tear during physical contact between two football players. The highest decrease in force at break for standard polyester fabrics with elastane was up to 26%. For recycled polyester fabrics, it was up to 15%. The results of the bursting force tests did not indicate the insufficient quality of the recycled fabrics compared to the standard fabrics. The average ability of the fabrics to absorb moisture decreased up to 23% after aging, while the wetting time increased up to 30%. The highest increase was observed for recycled fabrics. There is a strong negative correlation between the wetting time and moisture spreading speed of investigated fabrics. The loss of mass per unit area due to abrasion was greater for recycled polyester fabrics. This suggests that the emission of fibres into the environment caused by the abrasion process is greater for recycled polyester fabrics. The evidence from this study implies that there were neither chemical changes nor aging-related cracks on the surface of the fibres. The analysis presented provided insights for further research comparing the properties of recycled and standard polyester knitted fabrics. It has also contributed to a better understanding of the behaviour of sportswear materials due to aging, which is important to keep athletes performing at the highest level.

## Figures and Tables

**Table 1:** Fabric ID and description.

Fabric ID	PE92-197	PE87-141	P100-130	P100-185
Main raw material	92% PES standard	87% PES standard	100% PES recycled	100% PES recycled
Additional raw material	8% Elastane	13% Elastane	-	-
Mass per unit area	197 g/m^2^	141 g/m^2^	130 g/m^2^	185 g/m^2^

**Table 2:** Details of aging procedure.

Designation	Su Exposure	Duration, min	AS Exposure	Duration, min	Number of Wcyc	Duration, min
A0	-	-	-	-	-	-
A1	+	1440	+	1260	12	444
A2	+	4320	+	3780	36	1332

Legend: Su—sun, AS—artificial sweat, Wcyc—number of washing cycles.

**Table 3:** Correlation coefficients for moisture management indices.

	WTT	WTB	TAR	BAR	TSS	BSS
WTT	1.00000					
WTB	0.99285	1.00000				
TAR	−0.47946	−0.48508	1.00000			
BAR	−0.49266	−0.49469	0.98765	1.00000		
TSS	−0.86727	−0.88493	0.44280	0.48298	1.00000	
BSS	−0.85289	−0.87779	0.48066	0.51955	0.99401	1.00000

Marked correlations are significant at *p* < 0.5000. Legend WTT—wetting time top, WTB—wetting time bottom, TAR—absorption rate top, BAR—absorption rate bottom, TSS—spreading speed top, BSS—spreading speed bottom.

## Data Availability

Data are contained within the article.
